# Chronic intermittent hypobaric hypoxia ameliorates osteoporosis after spinal cord injury through balancing osteoblast and osteoclast activities in rats

**DOI:** 10.3389/fendo.2023.1035186

**Published:** 2023-05-09

**Authors:** Li Zhang, Yingchao Yin, Jialiang Guo, Lin Jin, Zhiyong Hou

**Affiliations:** Department of Orthopaedic Surgery, Third Hospital of Hebei Medical University, Shijiazhuang, Hebei, China

**Keywords:** spinal cord injury, bone loss, chronic intermittent hypobaric hypoxia, bone trabecula, rat

## Abstract

**Introduction:**

As a common complication of spinal cord injury (SCI), most SCI patients suffer from osteoporosis. In our previous study, chronic intermittent hypobaric hypoxia (CIHH) could promote bone fracture healing. We speculated that it may act a role in the progression of osteoporosis. The current study purposed to explore the role of CIHH in the osteoporosis triggered by SCI in rats.

**Methods:**

A SCI-induced SCI model was established by completed transection at T9-T10 spinal cord of Wistar rats. One week after SCI, the rats were conducted to CIHH treatment (PB = 404 mmHg, Po2 = 84 mmHg) 6 hours a day for continuously 7 weeks.

**Results:**

The results of X-radiography and Micro-CT assessment demonstrated that compared with sham rats, the areal bone mineral density (BMD), bone volume to tissue volume, volumetric BMD, trabecular thickness, trabecular number, and trabecular connectivity were decreased. Trabecular bone pattern factor, trabecular separation, as well as structure model index were increased at the distal femur and proximal tibia of SCI rats, which were effectively reversed by CIHH treatment. Histomorphometry showed that CIHH treatment increased bone formation of SCI rats, as evidenced by the increased osteoid formation, the decreased number and surface of TRAP-positive osteoclasts. Furthermore, ELISA and real time PCR results showed that the osteoblastogenesis-related biomarkers, such as procollagen type 1 N-terminal propeptide, osteocalcin in serum, as well as ALP and OPG mRNAs in bone tissue were decreased, while the osteoclastogenesis-related biomarkers, including scleorostin in serum and RANKL and TRAP mRNAs in bone tissue were increased in SCI rats. Importantly, the deviations of aforementioned biomarkers were improved by CIHH treatment. Mechanically, the protective effects of CIHH might be at least partly mediated by hypoxia-inducible factor-1 alpha (HIF-1α) signaling pathway.

**Conclusion:**

The present study testified that CIHH treatment ameliorates osteoporosis after SCI by balancing osteoblast and osteoclast activities in rats.

## 1 Introduction

SCI is one of the most serious injuries in clinic that leads to paralysis as well as long-term immobilization (1). As a common complication of SCI, most patients with SCI suffer from osteoporosis (1). SCI patients undergo biphasic bone loss including 50-70% lower cancellous bone mineral density and gradual 25-35% cortical bone loss within ten years of injury (2). The risk of fracture at nontraditional sites including distal femur and proximal tibia elevates more than 20 times due to the severe bone loss (2). Thus, there is an urgent need to develop a deeper understanding of the progression of SCI-induced osteoporosis and find novel targets for its treatment. Moreover, the common treatments for osteoporosis include anti-resorptive modulators and bisphosphonates, which mainly contribute to bone resorption, but not for bone regeneration (3). These agents might have bad side effects (such as thromboembolism and hypocalcaemia) that seriously affect the life quality and health of patients. To solve these problems, keeping the balance between bone regeneration and bone resorption is essential for the therapy of osteoporosis.

Chronic intermittent hypobaric hypoxia (CIHH) is a treatment of moderate hypoxia, which simulates the high altitude of normoxia interruption (4, 5). Accumulating evidences have testified that CIHH protects several organs against the ischemia/reperfusion-induced injuries (6–8). For example, the acute ischemia/reperfusion injury of skeletal muscle leads to deceased contraction tension, immune cell infiltration, inflammation and apoptosis in muscle. These changes were further relieved by CIHH treatment (6). Moreover, it has been reported that high blood pressure could be decreased by CIHH in spontaneously hypertensive rats, which is mediated by the improvement of vascular remodeling and the repression of inflammation (9). Recently, our study has shown that CIHH contributed to bone fracture healing of rats by the enhancement of biomechanical strength and bone formation through activating the hypoxia-inducible factor-1 (4). These studies prompted us that CIHH might play a role in the progression of osteoporosis. The purpose of this study is to explore the effect of CIHH on osteoporosis in rats with SCI. The underlying mechanism was then studied.

## 2 Materials and methods

### 2.1 Rats, spinal cord transaction and CIHH treatment

Wistar rats (male, eight-week-old) were purchased from Si pei fu Biotechnology Co., Ltd. (Beijing, China). Rats were cared with food and water ad libitum under a 12/12 hour light-dark cycle. The animal study was reviewed and approved by the Institutional Animal Care and Use Committee at Third Hospital of Hebei Medical University following The Guideline for the Care and Use of Laboratory Animals.

Rats were randomly divided into four groups as follows: sham operated group (Sham), CIHH treatment group (CIHH), spinal cord injury group (SCI), as well as SCI plus CIHH group (SCI+CIHH). For establishing SCI, after anaesthesia, the spinal cord of rats was transected at the interspace between the 9th and 10th vertebral bodies (10). Animals in sham groups underwent the same surgery as described above, but not for the cut of spinal cord. Herein, barometric pressure was lowered to the level that mimicked 5,000 m high-altitude (PB = 404 mmHg, Po2 = 84 mmHg) (5). After surgery for a week, rats received SCI and CIHH treatment were put into to a hypobaric chamber to get CIHH treatment, six hours a day for continuously seven weeks. The rats in CIHH groups underwent CIHH treatment for 7 weeks as same as rats in SCI+CIHH groups. After operation, animals received penicillin (CHINO Pharmaceutical Group, Shijiazhuang, China) for 3 days.

### 2.2 X-radiography and micro-CT assessment

Rats were sacrificed at 8-week post-surgery. Areal BMD (aBMD) of proximal tibial metaphysis, distal femoral metaphysis, and lumbar spine (L3-L5) was assessed by employing the dual-energy X-ray absorptiometer according to the previous study (10, 11).

The femurs and tibiae were resected for microcomputed tomography (micro-CT). After fixation, the images of bones were captured using the Micro-CT imager (Bruker-microCT, Antwerpen, Belgium). The picture resolution was 18 μm voxel. Two hundred sections between the areas were further measured. After reconstruction of the X-ray images, co-registration was achieved. Then CTAn software was carried out to morphometrically assess the trabecular bone cylinder. The 3D representations of bones were obtained with CTVox software. The bone parameters include trabecular bone volume over total volume (BV/TV, %), BMD, trabecular thickness (Tb.Th, mm), trabecular separation (Tb.Sp, mm), trabecular bone pattern factor (Tb.Pf), trabecular number (Tb.N, 1/mm), trabecular connectivity (Conn.D, 1/mm-3), as well as structural model index (SMI).

### 2.3 Histology staining

Von Kossa staining was performed to visualize mineralized tissue and osteoid formation. Briefly, after paraffin-embedding, sections were deparaffinized in xylene and then dehydrated. Next, sections were placed in the Von Kossa silver solution and exposed to strong light for 60 min. Eosin was used for counterstaining. Images were captured with the BX53 microscope (Olympus, Tokyo, Japan). The osteoid surface/bone surface (OS/BS) and mineralizing surface/bone surface (MS/BS) was quantified with Image-Pro Plus software.

Tartrate-resistant acid phosphatase (TRAP) staining was employed to observe the osteoclasts in distal femur slices. Briefly, a commercial TRAP assay kit (Sigma, MO, USA) was used as per the users’ instructions. Lastly, the pictures were taken by using the BX53 microscope. Resorption surface was calculated as follows: TRAP-positive (Oc.S)/total bone surface (BS). Osteoclast number was normalized with tissue area (N.Oc/T.Ar).

### 2.4 Serum measurements

Serum levels of sclerostin, osteocalcin, procollagen type 1 N-terminal propeptide (P1NP), and cross linked C-telopeptide of type I collagen (CTX-I) were measured using ELISA kits from USCN (Wuhan, China) and Fine Biotech (Wuhan, China) in accordance with the manufacturers’ protocols.

### 2.5 Quantitative real-time PCR

Total RNA was extracted from bone tissue of distal femur and further reversely transcribed to complementary DNA employing M-MLV reverse transcriptase (Tiangen). PCR primers were synthesized by GenScript Biotech (Nanjing, China). The amplification was carried out by employing the 2 × Taq MasterMix (BioTeke, Beijing, China) along with SYBR reagent (Solarbio, Beijing, China). GAPDH was used as the endogenous control, and the relative gene expression was estimated with the 2^-ΔΔCt^ method. The sequences of primers used in the current study were revealed in **Table 1**.

**Table 1:** The sequences of primers used for real-time PCR.

Gene	Sequences (forward)		Sequences (reverse)
ALP	CACGACAATCGGGATGAAC		GCCTTGACCACAGCACCTA
OPG	CATACCACTTTCCCAAAACCGTC		TCAACTGCCATTTCAAGAGCC
RANKL	ATGATGGAAGGTTCGTGGCT		AAGAGGACAGACTGACTTTATGGG
TRAP	GACGCCAATGACAAGAGGT		AAACGCAAACGGTAATAAGG
HIF-1α	CTATGTCGCTTTCTTGG		TTTCTGCTGCCTTGTAT
HO-1	CGAAACAAGCAGAACCCA		CACCAGCAGCTCAGGATG
VEGFα	GCTTTACTGCTGTACCTCCAC		ACGCACTCCAGGGCTTC

### 2.6 Western blot

Total protein was extracted from bone tissue of distal femur using RIPA lysis solution mixed with phenylmethanesulfonyl fluoride (100:1). The concentration of protein was detected with BCA Protein Assay Kit (Solarbio). Proteins were separated by 12% SDS-PAGE and then transferred to PVDF membranes. After blocked with 5% (M/V) skimmed milk, membranes were incubated with primary antibodies at 4°C overnight and secondary antibodies at 37°C for an hour. Subsequently, the probes were incubated with electrochemiluminescence reagent for 5 min, and the images were captured with Gel-Pro-Analyzer gel imaging system. The primary antibodies were as follows. Anti-hypoxia-inducible factor-1 alpha (HIF-1α; 1:500; Proteintech, Wuhan, China), anti-vascular endothelial growth factor alpha (VEGFα; 1:1000; Boster, Wuhan, China), anti-heme oxygenase-1 (HO-1; 1:500; Boster), and anti-GAPDH (1:10000; Proteintech). Secondary antibodies were as follows. Goat anti-rabbit HRP-conjugated IgG (1:3000; Solarbio) andgoat anti-mouse HRP-conjugated IgG (1:3000; Solarbio).

### 2.7 Statistical analysis

Results are represented as mean ± standard error (SD). Statistical analyses were performed by employing Graphpad Prism 8 software. Statistical comparisons among multiple groups were analyzed by one-way ANOVA followed by Tukey *post-hoc* analysis. *p* < 0.05 was considered statistically significant.

## 3 Results

### 3.1 Effects of CIHH on aBMD in SCI rats

**Figure 1:**
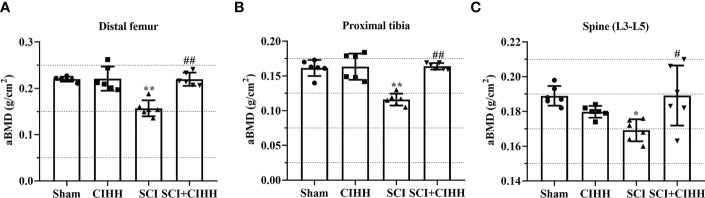
Effect of chronic intermittent hypobaric hypoxia (CIHH) on areal BMD (aBMD) in SCI rats. The spinal cord of rats was transected at the interspace between the 9th and 10th vertebral bodies, followed by a treatment of CIHH treatment (PB = 404 mmHg, Po2 = 84 mmHg) 6 hours a day for continuously 7 weeks. aBMD was acquired at distal femoral metaphysis **(A)**, proximal tibial metaphysis **(B)**, and lumbar spine (L3-L5) **(C)** in SCI rats. Sham: Sham operated group; SCI: Spinal cord injury group; CIHH: CIHH group; SCI+CIHH: SCI plus CIHH group. Data are expressed as mean ± SD. *n* = 6 in each group. ^*^ *p* < 0.05, ^**^ *p* < 0.01 *vs* Sham; ^#^ *p* < 0.05, ^##^ *p* < 0.01 *vs* SCI.

Firstly, the effects of CIHH on aBMD (the gold standard for osteoporosis) in SCI rat were investigated. As shown in **Figures 1A-C**, at 8-week after SCI injury, aBMD in distal femoral and proximal tibial metaphysis, and lumbar spine (L3–L5) was reduced compared to sham. The changes of aBMD was further elevated by CIHH treatment (P<0.05-0.01).

### 3.2 Effects of CIHH on trabecular bone architecture at the trabecular distal femur and proximal tibial metaphysis in SCI rats

**Figure 2:**
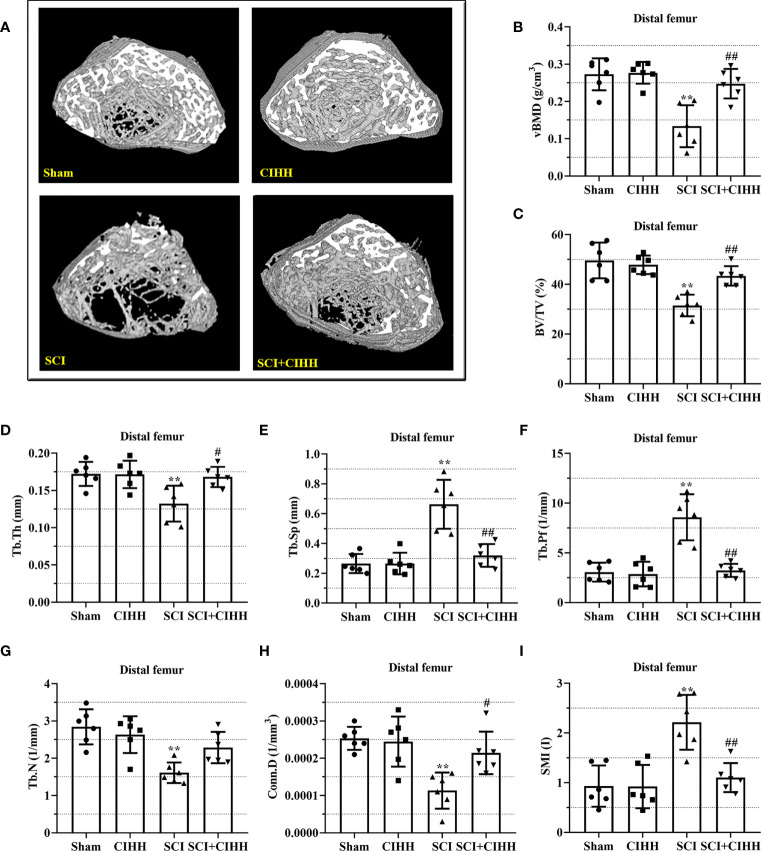
Effect of chronic intermittent hypobaric hypoxia (CIHH) on trabecular bone architecture at distal femoral metaphysis region in SCI rats. The spinal cord of rats was transected at the interspace between the 9th and 10th vertebral bodies, followed by a treatment of CIHH treatment (PB = 404 mmHg, Po2 = 84 mmHg) 6 hours a day for continuously 7 weeks. **(A)** Representative 3D images of trabecular microarchitecture; Representative parameters of trabecular bone architecture including **(B)** vBMD (g/cm-3); **(C)** Trabecular bone volume over total volume (BV/TV %); **(D)** Trabecular thickness (Tb.Th, mm); **(E)** trabecular separation (Tb.Sp, mm); **(F)** trabecular bone pattern factor (Tb.Pf, 1/mm); **(G)** trabecular number (Tb.N, 1/mm); **(H)** Trabecular connectivity (Conn.D, 1/mm-3); **(I)** structural model index (SMI, range from 0 to 3, with 0 = platelike and 3 = rodlike) at distal femoral metaphysis region of SCI rats. Sham: Sham operated group; SCI: Spinal cord injury group; CIHH: CIHH group; SCI+CIHH: SCI plus CIHH group. Data are expressed as mean ± SD. *n* = 6 in each group. ^**^ *p* < 0.01 *vs* Sham; ^#^ *p* < 0.05, ^##^ *p* < 0.01 *vs* SCI.

**Figure 3:**
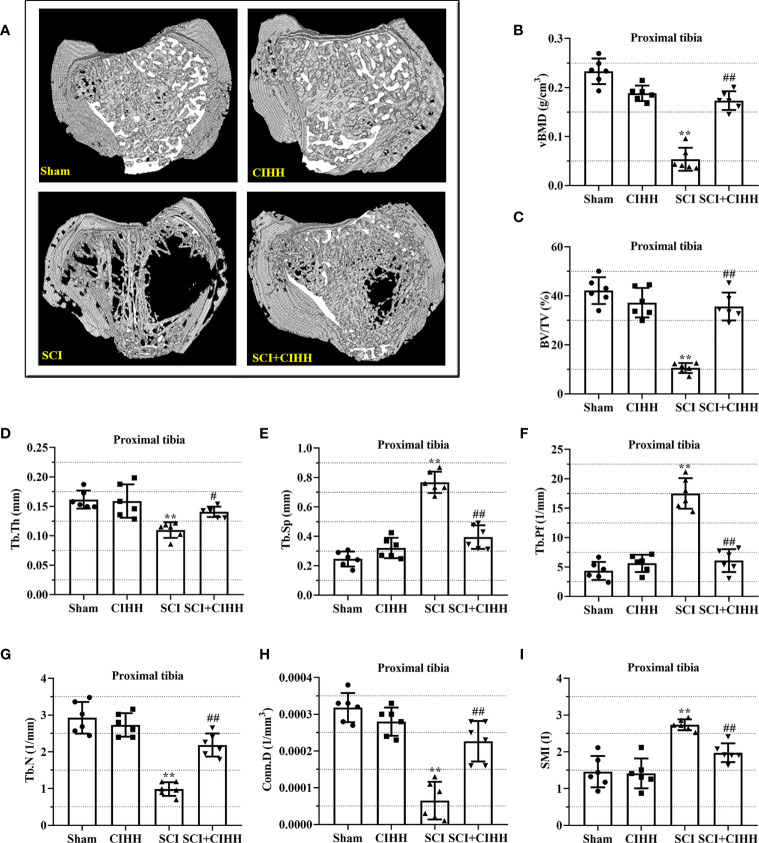
Effect of chronic intermittent hypobaric hypoxia (CIHH) on trabecular bone architecture at proximal tibial metaphysis region in SCI rats. The spinal cord of rats was transected at the interspace between the 9th and 10th vertebral bodies, followed by a treatment of CIHH treatment (PB = 404 mmHg, Po2 = 84 mmHg) 6 hours a day for continuously 7 weeks. **(A)** Representative 3D images of trabecular microarchitecture; Representative parameters of trabecular bone architecture including **(B)** vBMD (g/cm-3); **(C)** Trabecular bone volume over total volume (BV/TV %); **(D)** Trabecular thickness (Tb.Th, mm); **(E)** trabecular separation (Tb.Sp, mm); **(F)** trabecular bone pattern factor (Tb.Pf, 1/mm); **(G)** trabecular number (Tb.N, 1/mm); **(H)** Trabecular connectivity (Conn.D, 1/mm-3); **(I)** structural model index (SMI, range from 0 to 3, with 0 = platelike and 3 = rodlike) at proximal tibial metaphysis region of SCI rats. Sham: Sham operated group; SCI: Spinal cord injury group; CIHH: CIHH group; SCI+CIHH: SCI plus CIHH group. Data are expressed as mean ± SD. *n* = 6 in each group. ^**^ *p* < 0.01 *vs* Sham; ^#^ *p* < 0.05, ^##^ *p* < 0.01 *vs* SCI.

The changes of bone architecture were further examined by employing the high-resolution µCT. SCI rats exhibited bone growth retardation and severe osteopenia, which was mitigated by CIHH treatment (**Figure 2A**). CIHH treatment reversed the SCI-induced reduction of vBMD, BV/TV, Tb.Th and Tb.N, as well as the elevation of Tb.Sp and Tb.Pf (P<0.05-0.01, **Figures 2B-G**). Also, the decrease of Conn. D and increase of SMI in SCI rats were reverted by CIHH (P<0.05-0.01, **Figures 2H, I**). The change of bone architecture at the proximal tibial metaphysis was also examined and similar result like distal femoral metaphysis was acquired (**Figures 3A-I**). The result indicates that CIHH treatment prevents bone loss post SCI.

### 3.3 Effects of CIHH on the bone formation in SCI rats

**Figure 4:**
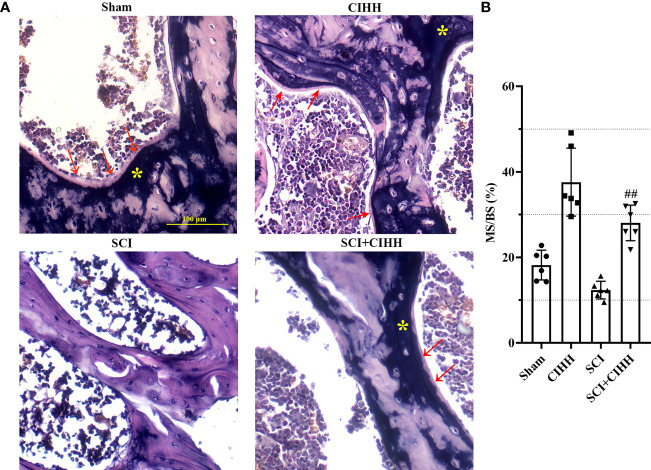
Von Kossa staining at trabecular bone of distal femur. The spinal cord of rats was transected at the interspace between the 9th and 10th vertebral bodies, followed by a treatment of CIHH treatment (PB = 404 mmHg, Po2 = 84 mmHg) 6 hours a day for continuously 7 weeks. **(A)** Photomicrographs of metaphyseal trabecular bone stained with von Kossa (for mineral, yellow stain, asterisk; for unmineralized bone matrix, red arrows; magnification ×200). Scale bar is 100 μm. **(B)** MS/BS (mineralizing surface/bone surface) assessed by quantification of Von Kossa staining. Sham: Sham operated group; SCI: Spinal cord injury group; CIHH: CIHH group; SCI+CIHH: SCI plus CIHH group. Data are expressed as mean ± SD. *n* = 6 in each group. ^##^ *p* < 0.01 *vs* SCI.

We then explored the effects of CIHH on bone formation in SCI rats. Von Kossa staining was carried out to detect calcification and assess the bone formation of distal femur. The results revealed that the MS/BS was elevated by CIHH treatment in the femur tissues of both SCI and sham rats, indicating the enhancement of bone mineralization by CIHH (**Figures 4A, B**). The similar changes were obtained in OS/BS (**Supplemental Figure 1**). These results suggested that CIHH treatment promotes bone formation.

**Figure 5:**
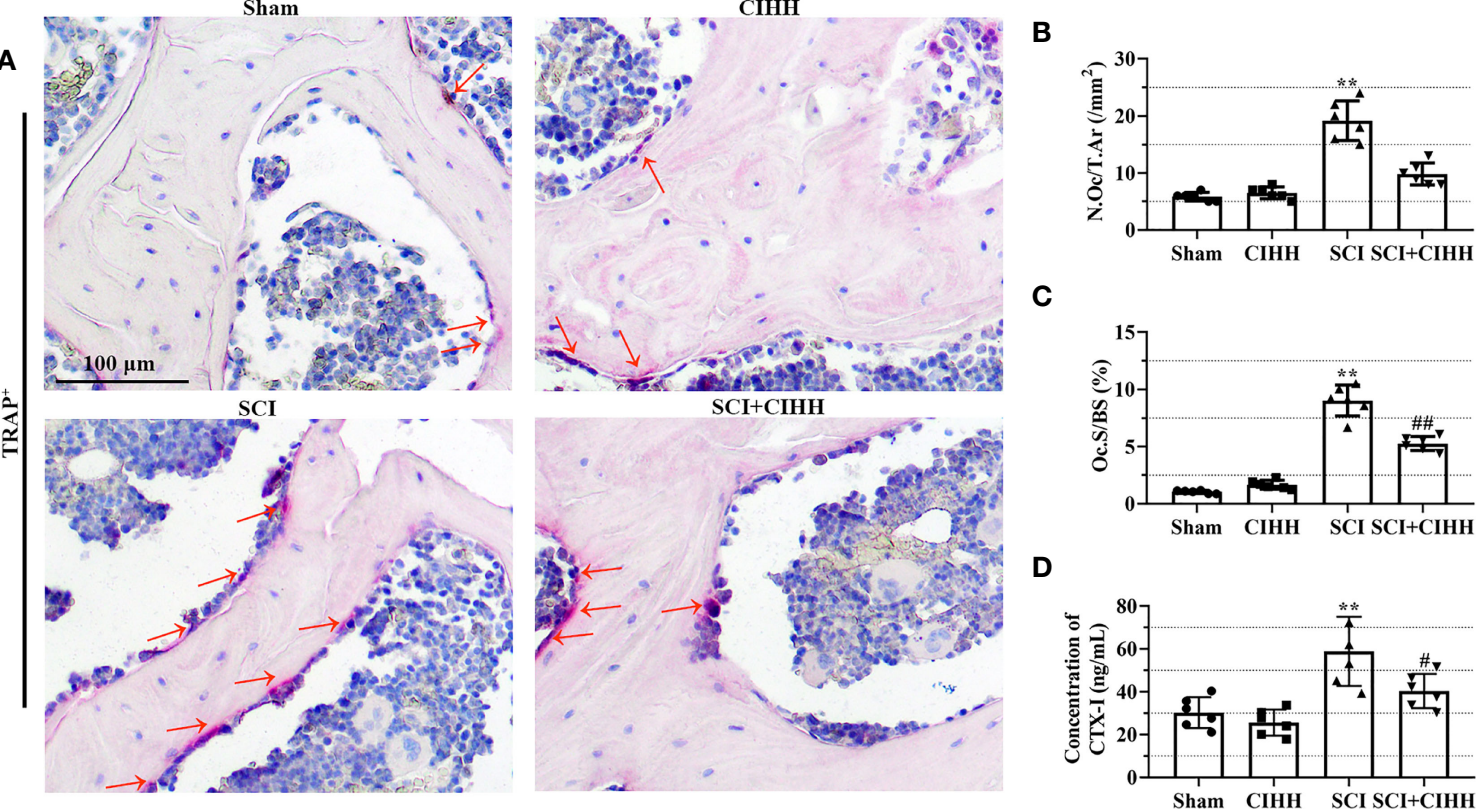
Effects of chronic intermittent hypobaric hypoxia (CIHH) on osteoclast differentiation and bone resorption markers. The spinal cord of rats was transected at the interspace between the 9th and 10th vertebral bodies, followed by a treatment of CIHH treatment (PB = 404 mmHg, Po2 = 84 mmHg) 6 hours a day for continuously 7 weeks. **(A)** Representative immunohistochemical images for tartrate-resistant acid phosphatase (TRAP; magnification ×200) at trabecular bone of distal femur. Red arrows indicate TRAP-positive cells. Scale bar is 100 μm. **(B)** Osteoclast number (N.Oc/T.Ar). **(C)** Osteoclast surfaces (Oc.S/BS). **(D)** The concentration of cross linked C-telopeptide of type I collagen (CTX-I) in the serum of rats. Sham: Sham operated group; SCI: Spinal cord injury group; CIHH: CIHH group; SCI+CIHH: SCI plus CIHH group. Data are expressed as mean ± SD. *n* = 6 in each group. ^**^ *p* < 0.01 *vs* Sham; ^#^ *p* < 0.05, ^##^ *p* < 0.01 *vs* SCI.

TRAP staining was performed to identify osteoclast differentiation. There was a trend that the number and surface of TRAP-positive osteoclasts were increased in SCI rats (P<0.01), which were inhibited by CIHH treatment (P<0.01, **Figures 5A-C**). Moreover, the serum samples collected from the sacrificed rats were analyzed from the presence of bone resorption marker CTX-I. The results demonstrated that CIHH reduced the SCI-induced elevation of CTX-I concentration in the serum of rats (**Figure 5D**), which reflected the inhibition of bone resorption activity. The data indicates that CIHH inhibits osteoclast differentiation in SCI rats.

### 3.4 Effects of CIHH on the expression of genes and proteins involved in osteoblastogenesis and osteoclastogenesis in the serum and femurs in SCI rats

**Figure 6:**
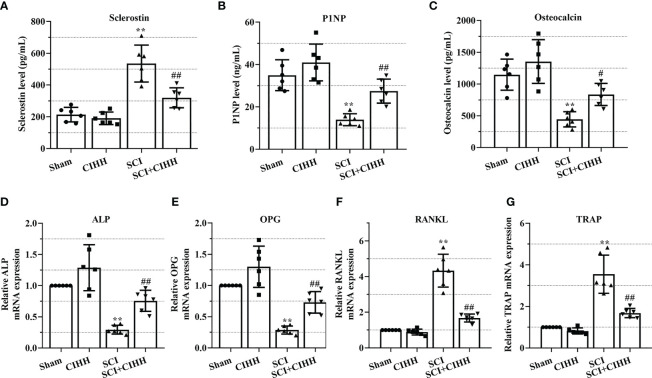
Effects of chronic intermittent hypobaric hypoxia (CIHH) on the expression level of indicators for osteoblastogenesis and osteoclastogenesis. The spinal cord of rats was transected at the interspace between the 9th and 10th vertebral bodies, followed by a treatment of CIHH treatment (PB = 404 mmHg, Po2 = 84 mmHg) 6 hours a day for continuously 7 weeks. The concentration of **(A)** sclerostin, **(B)** procollagen type 1 N-terminal propeptide (P1NP), and **(C)** osteocalcin in serum of rats were detected by ELISA. Relative mRNA expression of **(D)** alkaline phosphatase (ALP), **(E)** osteoprotegerin (OPG), **(F)** receptor activator of NF-κB ligand (RANKL), and **(G)** tartrate-resistant acid phosphatase (TRAP) in distal femur of rats was assessed by real-time PCR. Sham: Sham operated group; SCI: Spinal cord injury group; CIHH: CIHH group; SCI+CIHH: SCI plus CIHH group. Data are expressed as mean ± SD. *n* = 6 in each group. ^**^ *p* < 0.01 *vs* Sham; ^#^ *p* < 0.05, ^##^ *p* < 0.01 *vs* SCI.

The role of CIHH in the levels of genes and protein involved in osteoblastogenesis and osteoclastogenesis in the serum and femurs in SCI rats was investigated. Serum osteocalcin, P1NP, sclerostin, and the biomarkers involved in osteoblastogenesis and osteoclastogenesis were detected by employing the ELISA. As revealed in **Figures 6A-C**, the levels of P1NP and osteocalcin were decreased and sclerostin concentration was elevated in SCI rats than that of sham groups. The changes of these markers were further reversed by CIHH treatment (P<0.05-0.01).

Also, the relative levels of osteoblastogenesis and osteoclastogenesis-related genes were further detected by using real-time PCR. As revealed in **Figures 6D-G**, the SCI-induced downregulation of ALP and OPG, as well as the upregulation of RANKL and TRAP were rescued by CIHH. These findings suggest that CIHH maintains the balance between osteoblast and osteoclast activities.

### 3.5 Effects of CIHH on the expression of hypoxia and bone vasculature-related gene and protein

**Figure 7:**
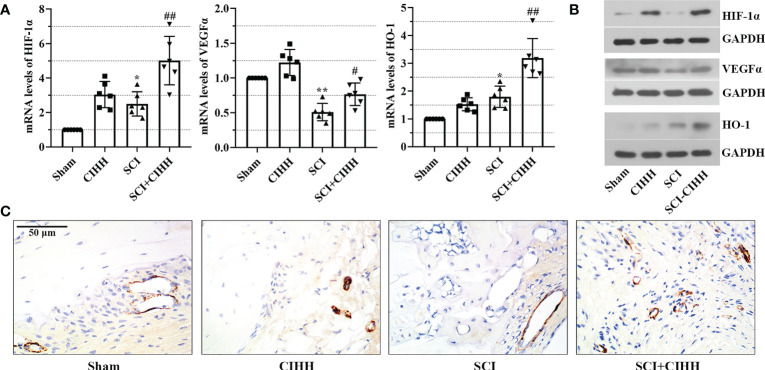
Effects of CIHH treatment on the expression of hypoxia and the bone vasculature-related gene and protein. The spinal cord of rats was transected at the interspace between the 9th and 10th vertebral bodies, followed by a treatment of CIHH treatment (PB = 404 mmHg, Po2 = 84 mmHg) 6 hours a day for continuously 7 weeks. **(A, B)** mRNA and protein levels of hypoxia-inducible factor-1 alpha (HIF-1α), vascular endothelial growth factor alpha (VEGFα), and heme oxygenase-1 (HO-1) in distal femur of rats were measured by real-time PCR and western blot, respectively. **(C)** The expression of CD31 was detected with immunohistochemistry assay (magnification ×400). Scale bar is 50 μm. Sham: Sham operated group; SCI: Spinal cord injury group; CIHH: CIHH group; SCI+CIHH: SCI plus CIHH group. Data are expressed as mean ± SD. n = 6 in each group. *p < 0.05, **p < 0.01 vs Sham; ^#^ *p* < 0.05, ^##^ *p* < 0.01 vs SCI.

Since CIHH is a treatment of moderate hypoxia. HIF1α is a vital hypoxia-induced transcription factor that is linked with fracture repair and SCI injury. Thus, we considered the protective function of CIHH might be associated with HIF1α and its downstream factors. The expression of these factors including HIF-1α, VEGFα, and HO-1 was evaluated by real time PCR and western blot. As shown in **Figures 7A, B**, the mRNA and protein expression of HIF-1α, VEGFα, and HO-1 were up-regulated by CIHH treatment in distal femur of rat with SCI. There was a trend that CIHH increased the SCI-induced reduction of CD31 expression, which was detected by immunohistochemistry assay (**Figure 7C**). The data implied that CIHH relieved the SCI-induced osteoporosis might be at least partly mediated by the HIF-1α signaling pathway.

## 4 Discussion

Clinically, bone fragility is divided into primary (induced by menopause or aging) and secondary osteoporosis (induced by diseases, drugs, or factors), and SCI-induced osteoporosis belongs to the latter. Recently, many studies focus on estrogen depletion and aging-induced osteoporosis (12, 13), whereas there are limited data in osteoporosis induced by SCI. It has been reported that SCI causes more severe bone loss and microstructure deterioration compared with ovariectomy and sciatic neurectomy (which triggers disuse osteoporosis) (14, 15). We considered whether CIHH acted as a role in SCI-induced osteoporosis. Thus, SCI-osteoporosis was chosen in this study. Herein, the dual energy X-ray result showed that aBMD, the gold standard for osteoporosis, was decreased in femur, tibia, and lumbar spine of SCI rats. The µCT results revealed that BMD, BV/TV, Tb.Th, Tb.N, and Conn. D were reduced, while Tb.Sp, Tb.Pf as well as SMI were elevated in femur and tibia in rats with SCI. The results of Von Kossa staining showed that bone mineralization was reduced in femur of SCI rats. These results confirmed osteoporosis induced by SCI. Importantly, all deviations of bone morphological structure in SCI rats were effectively reversed or prevented by CIHH treatment. These findings imply that CIHH exerts anti-osteoporosis function in SCI rats.

Although acute and long-term secondary complications are more common in SCI patients, chronic complications seriously affect the life health of patients (16). Thus, the prevention and therapy of chronic secondary complications in SCI patients is of great significance to ameliorate the life quality of suffers. Osteoporosis is an important chronic complication of SCI. Previous reports have shown that rapid osteoporosis occurs in SCI patients (17), and chronic motor-complete SCI experiences severe bone loss at 3-16 weeks after SCI in rodents (18, 19). It is an urgent need to develop effective methods for clinical treatment post SCI, however, at present, few therapeutic strategies are available to relieve the complications of this disease (20). In this study, CIHH was found to promote bone formation and prevent deterioration of trabecular bone at femur and tibia in SCI rat. Moreover, there are other complications including chronic liver pathology (21), vascular dysfunction (22), ventricular arrhythmias (23), and dysfunction of cardiac (24), respiratory (25), and nervous systems (26). Unfortunately, the role of CIHH on these complications is still unknown. Lack of the investigation of CIHH on these complications is a limitation of this study. Notably, the current pattern of CIHH used in our study has been reported to exert protective function in renovascular hypertension (27), cardiac dysfunction (28), arrhythmia (29), cerebral ischemia (8), seizures (30), endoplasmic reticulum stress-induced liver damage (31), and diabetic nephropathy (32). The effects of CIHH on respiratory system disease remain unclear, whereas it has been testified that CIHH enhances sympatho-respiratory coupling, which reflects a reciprocal interaction between autonomic and respiratory control systems (33).These studies imply the potential of CIHH treatment on these complication-induced by SCI.

Sclerostin could destroy the bone homeostasis by the repression of osteogenesis upon osteoblasts regulation (34). Also, sclerostin could facilitate the resorptive function of osteoclasts (35). Bone loss and the up-regulation of sclerostin in osteocytes are found in rodent model with SCI (36). On the contrary, sclerostin depletion can cause diseases including sclerosteosis as well as Van Buchem’s disease (37). PINP has been found to reflect histomorphometric measure of bone formation, and has been identified as the most promising marker of bone formation in osteoporosis (38, 39). Osteocalcin is one of the key players in bone endocrinology. It is a product of osteoblasts and is considered a marker of osteogenesis (40). These studies indicate the vital role of sclerostin, PINP, osteocalcin in bone homeostasis in osteoporosis induced by SCI. In this study, serum PINP and osteocalcin were decreased and sclerostin was increased in SCI rats, and the change of these factors was effectively reverted, which suggests that CIHH enhances osteogenesis and reduces bone resorption, resulting in the improvement of osteoporosis.

The imbalance between osteoblasts and osteoclasts is recognized as a major cause of bone loss or osteoporosis (41). Osteoblasts and osteoclasts act as major parts in regulating the bone homeostasis by exerting functions in bone formation and bone resorption, respectively (42). Association between the two processes is critical for keeping the bone homeostasis (43). It has been reported that the bone lost after SCI arises in two periods. In the first period, bone is rapidly reabsorbed and further stabilized. In the second period, there are bone loss and osteogenesis repression (44). In this study, results from TRAP staining assay showed that CIHH decreased the elevated the number and surface of osteoclast in distal femur sections post SCI. CIHH reduced the SCI-induced elevation of CTX-I concentration in the serum of rats. The levels of TRAP in serum could better reflect the inhibition of bone resorption activity. The lack of the detection is another limitation of this study. All these data illustrate the inhibitory effect of CIHH on bone resorption by the regulation of osteoclasts *in vivo*. Given the observation of the function of CIHH *in vivo* SCI model, it is possible that CIHH might act as a similar role *in vitro*. It has been reported that hypoxia inhibited osteoclast differentiation of BMM (bone marrow-derived macrophages) and RAW264.7 cells (mouse leukemic monocyte/macrophage cell line) (45). HIF1α in osteoblast restrains the osteoclastogenesis of BMM *in vitro* (46). The studies indicate the potential of hypoxia and HIF1α in the regulation of osteoclastogenesis. Based on the results of CIHH upregulated the expression of HIF1α, it was implied that CIHH might act as a repressed role in the osteoclastogenesis of BMM *in vitro*. Lack of the cell experiment is the third limitation of this study, which will be completed in our further investigation.

Bone formation is a two-step process, with the osteoblasts laying down osteoid, which is further mineralized. Briefly, after bone matrix is deposited by osteoblasts, cross-linking of collagen fibers and other changes occur to prepare the matrix for mineralization. Due to these necessary changes, collectively known as maturation, there is both spatial and temporal separation between matrix and mineral apposition that is presented as osteoid bone (47). Thus, we speculated that CIHH might enhance the osteoid formation of SCI rats, which might then contribute to the bone mineralization and ultimately promoted bone formation. Similar to the results, it has been reported that SCI leads to a marginal decrease of osteoid volume and osteoid surface at trabecular bone of distal femur, and the recovery of SCI is accompanied with the elevation of osteoid and mineralized bone formation (48). The histomorphometric finding is in line with our ELISA results in which the SCI-induced reduction of serum osteocalcin level was increased by CIHH treatment, indicating the promotion of bone formation. Thus, we preferred that CIHH promoted the unmineralized bone and mineralized bone might be appropriate.

HIF1α is a vital hypoxia-induced transcription factor, and its high expression is linked with the exercise function recovery after SCI (49). During hypoxia, HIF-1α stably exists and is able to regulate the transcription of downstream factors including VEGFα and HO-1, which contribute to angiogenesis, anti-inflammatory/antioxidant, and the improvement of locomoter ability in fracture repair and SCI injury (50, 51). CIHH is a treatment of moderate hypoxia. We speculated that the protective effects of CIHH on osteoporosis induced by SCI might be associated with the regulation of HIF-1α and its downstream factors. Herein, there was a trend that CIHH treatment up-regulated the SCI-induced decrease of VEGFα and CD31 in distal femur of rat with SCI. Consistent with our findings, Ding et al. have proven that SCI-injured mice exhibit reduced levels of intraosseous blood vessel parameters (52, 53). Theoretically, vasculature disruption may lead to local hypoxia in bone microenvironment after injury (50). In this study, there were no marked changes of HIF-1α protein expression between control and SCI rat. We preferred that this may due to the short half-life of HIF-1α. It stably exists in the environment with oxygen content less than 5%. Once normal oxygen restores, HIF-1α has a half-life less than five minutes (50). Herein, it is possible that HIF-1α had been returned to baseline levels at seven week post SCI. Moreover, HO-1 is a cytoprotective enzyme that responds to oxidative or inflammatory stimuli (54). The expression of HO-1 may be compensatory increased in distal femur post-SCI, which was further up-regulated by CIHH. Interestingly, HO-1 and VEGFα are the downstream factors of HIF-1α, whose expression has no marked changes. HO-1 and VEGFα may be regulated by multiple pathways due to the complex pathological environment of SCI. Thus, we preferred that CIHH protected against SCI-induced osteoporosis might be at least partly mediated by the upregulation of HIF-1α signaling pathway.

## 5 Conclusion

In conclusion, CIHH ameliorates osteoporosis after SCI through balancing osteoblast and osteoclast activities in rats. The results of this study expand the application of CIHH and provide a basic investigation for the therapy of SCI-associated osteoporosis.

## Data availability statement

The original contributions presented in the study are included in the article/**Supplementary Material**. Further inquiries can be directed to the corresponding author.

## Ethics statement

The animal study was reviewed and approved by Institutional Animal Care and Use Committee at Third Hospital of Hebei Medical University.

## Author contributions

The authors confirm contribution to the paper as follows: study conception and design: LZ and ZH. Data collection: YY; analysis and interpretation of results. LZ, JG and LJ; draft manuscript preparation. LZ and ZH. All authors made a significant contribution to the work reported and read the final article and approved its submission.

## References

[1] Shams R Drasites KP Zaman V Matzelle D Shields DC Garner DP. The pathophysiology of osteoporosis after spinal cord injury. Int J Mol Sci (2021) 22(6):3057. doi: 10.3390/ijms22063057 33802713 PMC8002377

[2] Otzel DM Conover CF Ye F Phillips EG Bassett T Wnek RD. Longitudinal examination of bone loss in Male rats after moderate-severe contusion spinal cord injury. Calcified Tissue Int (2019) 104:79–91. doi: 10.1007/s00223-018-0471-8 PMC8349506 30218117

[3] Patel D Wairkar S. Bone regeneration in osteoporosis: Opportunities and challenges. Drug delivery Trans Res (2022) 13(2):419–32. doi: 10.1007/s13346-022-01222-6 35994158

[4] Zhang L Jin L Guo J Bao K Hu J Zhang Y. Chronic intermittent hypobaric hypoxia enhances bone fracture healing. Front Endocrinol (Lausanne) (2020) 11:582670. doi: 10.3389/fendo.2020.582670 33664707 PMC7921462

[5] Huang YJ Yuan YJ Liu YX Zhang MY Zhang JG Wang TC. Nitric oxide participates in the brain ischemic tolerance induced by intermittent hypobaric hypoxia in the hippocampal Ca1 subfield in rats. Neurochem Res (2018) 43:1779–90. doi: 10.1007/s11064-018-2593-9 29995175

[6] Cheng WJ Liu X Zhang L Guo XQ Wang FW Zhang Y. Chronic intermittent hypobaric hypoxia attenuates skeletal muscle ischemia-reperfusion injury in mice. Life Sci (2019) 231:116533. doi: 10.1016/j.lfs.2019.06.008 31173783

[7] Ma HJ Li Q Ma HJ Guan Y Shi M Yang J. Chronic intermittent hypobaric hypoxia ameliorates Ischemia/Reperfusion-induced calcium overload in heart *Via* Na/Ca2+ exchanger in developing rats. Cell Physiol Biochem (2014) 34:313–24. doi: 10.1159/000363001 25096990

[8] Wang J Zhang S Ma H Yang S Liu Z Wu X. Chronic intermittent hypobaric hypoxia pretreatment ameliorates ischemia-induced cognitive dysfunction through activation of Erk1/2-Creb-Bdnf pathway in anesthetized mice. Neurochem Res (2017) 42:501–12. doi: 10.1007/s11064-016-2097-4 27822668

[9] Chen H Yu B Guo X Hua H Cui F Guan Y. Chronic intermittent hypobaric hypoxia decreases high blood pressure by stabilizing the vascular renin-angiotensin system in spontaneously hypertensive rats. Front Physiol (2021) 12:639454. doi: 10.3389/fphys.2021.639454 33841179 PMC8024534

[10] Sun L Pan J Peng Y Wu Y Li J Liu X. Anabolic steroids reduce spinal cord injury-related bone loss in rats associated with increased wnt signaling. J spinal cord Med (2013) 36:616–22. doi: 10.1179/2045772312y.0000000020 PMC3831322 24090150

[11] Bramlett HM Dietrich WD Marcillo A Mawhinney LJ Furones-Alonso O Bregy A. Effects of low intensity vibration on bone and muscle in rats with spinal cord injury. Osteoporosis Int (2014) 25:2209–19. doi: 10.1007/s00198-014-2748-8 24861907

[12] Gjoksi B Ghayor C Siegenthaler B Ruangsawasdi N Zenobi-Wong M Weber FE. The epigenetically active small chemical n-methyl pyrrolidone (Nmp) prevents estrogen depletion induced osteoporosis. Bone (2015) 78:114–21. doi: 10.1016/j.bone.2015.05.004 25959414

[13] Luo D Li J Chen K Rong X Guo J. Untargeted metabolomics reveals the protective effect of fufang zhenshu tiaozhi (Ftz) on aging-induced osteoporosis in mice. Front Pharmacol (2018) 9:1483. doi: 10.3389/fphar.2018.01483 30670964 PMC6331458

[14] Jiang SD Shen C Jiang LS Dai LY. Differences of bone mass and bone structure in osteopenic rat models caused by spinal cord injury and ovariectomy. Osteoporosis Int (2007) 18:743–50. doi: 10.1007/s00198-006-0299-3 17216554

[15] Liu D Zhao CQ Li H Jiang SD Jiang LS Dai LY. Effects of spinal cord injury and hindlimb immobilization on sublesional and supralesional bones in young growing rats. Bone (2008) 43:119–25. doi: 10.1016/j.bone.2008.03.015 18482879

[16] Guilcher SJT Everall AC Patel T Packer TL Hitzig SL Lofters AK. Medication adherence for persons with spinal cord injury and dysfunction from the perspectives of healthcare providers: A qualitative study. J spinal cord Med (2019) 42:215–25. doi: 10.1080/10790268.2019.1637644 PMC6781202 31573463

[17] Hatefi M Ahmadi MRH Rahmani A Dastjerdi MM Asadollahi K. Effects of curcumin on bone loss and biochemical markers of bone turnover in patients with spinal cord injury. World Neurosurg (2018) 114:e785–e91. doi: 10.1016/j.wneu.2018.03.081 29567290

[18] Beggs LA Ye F Ghosh P Beck DT Conover CF Balaez A. Sclerostin inhibition prevents spinal cord injury-induced cancellous bone loss. J Bone miner Res (2015) 30:681–9. doi: 10.1002/jbmr.2396 PMC8367350 25359699

[19] Voor MJ Brown EH Xu Q Waddell SW Burden RL Jr. Burke DA. Bone loss following spinal cord injury in a rat model. J neurotrauma (2012) 29:1676–82. doi: 10.1089/neu.2011.2037 PMC3353757 22181016

[20] Rodriguez G Berri M Lin P Kamdar N Mahmoudi E Peterson MD. Musculoskeletal morbidity following spinal cord injury: A longitudinal cohort study of privately-insured beneficiaries. Bone (2021) 142:115700. doi: 10.1016/j.bone.2020.115700 33091639 PMC9671069

[21] Sauerbeck AD Laws JL Bandaru VV Popovich PG Haughey NJ McTigue DM. Spinal cord injury causes chronic liver pathology in rats. J neurotrauma (2015) 32:159–69. doi: 10.1089/neu.2014.3497 PMC4298754 25036371

[22] Popa C Popa F Grigorean VT Onose G Sandu AM Popescu M. Vascular dysfunctions following spinal cord injury. J Med Life (2010) 3:275–85. PMC3019008 20945818

[23] Collins HL Rodenbaugh DW DiCarlo SE. Spinal cord injury alters cardiac electrophysiology and increases the susceptibility to ventricular arrhythmias. Prog Brain Res (2006) 152:275–88. doi: 10.1016/s0079-6123(05)52018-1 16198707

[24] Fossey MPM Balthazaar SJT Squair JW Williams AM Poormasjedi-Meibod MS Nightingale TE. Spinal cord injury impairs cardiac function due to impaired bulbospinal sympathetic control. Nat Commun (2022) 13:1382. doi: 10.1038/s41467-022-29066-1 35296681 PMC8927412

[25] Galeiras Vázquez R Rascado Sedes P Mourelo Fariña M Montoto Marqués A Ferreiro Velasco ME. Respiratory management in the patient with spinal cord injury. BioMed Res Int (2013) 2013:168757. doi: 10.1155/2013/168757 24089664 PMC3781830

[26] Karlsson AK. Autonomic dysfunction in spinal cord injury: Clinical presentation of symptoms and signs. Prog Brain Res (2006) 152:1–8. doi: 10.1016/s0079-6123(05)52034-x 16198689

[27] Guan Y Li N Tian YM Zhang L Ma HJ Maslov LN. Chronic intermittent hypobaric hypoxia antagonizes renal vascular hypertension by enhancement of vasorelaxation *Via* activating bkca. Life Sci (2016) 157:74–81. doi: 10.1016/j.lfs.2016.05.028 27216772

[28] Zhou JJ Wei Y Zhang L Zhang J Guo LY Gao C. Chronic intermittent hypobaric hypoxia prevents cardiac dysfunction through enhancing antioxidation in fructose-fed rats. Can J Physiol Pharmacol (2013) 91:332–7. doi: 10.1139/cjpp-2012-0059 23656204

[29] Zhou JJ Ma HJ Liu Y Guan Y Maslov LN Li DP. The anti-arrhythmic effect of chronic intermittent hypobaric hypoxia in rats with metabolic syndrome induced with fructose. Can J Physiol Pharmacol (2015) 93:227–32. doi: 10.1139/cjpp-2014-0343 25563803

[30] Zhen JL Wang WP Zhou JJ Qu ZZ Fang HB Zhao RR. Chronic intermittent hypoxic preconditioning suppresses pilocarpine-induced seizures and associated hippocampal neurodegeneration. Brain Res (2014) 1563:122–30. doi: 10.1016/j.brainres.2014.03.032 24680745

[31] Yuan F Teng X Guo Z Zhou JJ Zhang Y Wang S. Chronic intermittent hypobaric hypoxia ameliorates endoplasmic reticulum stress mediated liver damage induced by fructose in rats. Life Sci (2015) 121:40–5. doi: 10.1016/j.lfs.2014.11.019 25476828

[32] Tian YM Guan Y Li N Ma HJ Zhang L Wang S. Chronic intermittent hypobaric hypoxia ameliorates diabetic nephropathy through enhancing Hif1 signaling in rats. Diabetes Res Clin Pract (2016) 118:90–7. doi: 10.1016/j.diabres.2016.06.021 27351799

[33] Dick TE Hsieh YH Dhingra RR Baekey DM Galán RF Wehrwein E. Cardiorespiratory coupling: Common rhythms in cardiac, sympathetic, and respiratory activities. Prog Brain Res (2014) 209:191–205. doi: 10.1016/b978-0-444-63274-6.00010-2 24746049 PMC4052709

[34] van Bezooijen RL Roelen BA Visser A van der Wee-Pals L de Wilt E Karperien M. Sclerostin is an osteocyte-expressed negative regulator of bone formation, but not a classical bmp antagonist. J Exp Med (2004) 199:805–14. doi: 10.1084/jem.20031454 PMC2212719 15024046

[35] Wijenayaka AR Kogawa M Lim HP Bonewald LF Findlay DM Atkins GJ. Sclerostin stimulates osteocyte support of osteoclast activity by a rankl-dependent pathway. PloS One (2011) 6:e25900. doi: 10.1371/journal.pone.0025900 21991382 PMC3186800

[36] Metzger CE Gong S Aceves M Bloomfield SA Hook MA. Osteocytes reflect a pro-inflammatory state following spinal cord injury in a rodent model. Bone (2019) 120:465–75. doi: 10.1016/j.bone.2018.12.007 30550849

[37] Balemans W Van Hul W. Human genetics of sost. J musculoskeletal neuronal Interact (2006) 6:355–6. 17185822

[38] Chavassieux P Portero-Muzy N Roux JP Garnero P Chapurlat R. Are biochemical markers of bone turnover representative of bone histomorphometry in 370 postmenopausal women? J Clin Endocrinol Metab (2015) 100:4662–8. doi: 10.1210/jc.2015-2957 26505821

[39] Vasikaran S Eastell R Bruyère O Foldes AJ Garnero P Griesmacher A. Markers of bone turnover for the prediction of fracture risk and monitoring of osteoporosis treatment: A need for international reference standards. Osteoporosis Int (2011) 22:391–420. doi: 10.1007/s00198-010-1501-1 21184054

[40] Komori T. Functions of osteocalcin in bone, pancreas, testis, and muscle. Int J Mol Sci (2020) 21(20):7513. doi: 10.3390/ijms21207513 33053789 PMC7589887

[41] Kim JM Lin C Stavre Z Greenblatt MB Shim JH. Osteoblast-osteoclast communication and bone homeostasis. Cells (2020) 9(9):2073. doi: 10.3390/cells9092073 32927921 PMC7564526

[42] Boyle WJ Simonet WS Lacey DL. Osteoclast differentiation and activation. Nature (2003) 423:337–42. doi: 10.1038/nature01658 12748652

[43] Chen X Wang Z Duan N Zhu G Schwarz EM Xie C. Osteoblast-osteoclast interactions. Connective Tissue Res (2018) 59:99–107. doi: 10.1080/03008207.2017.1290085 PMC5612831 28324674

[44] Tan CO Battaglino RA Morse LR. Spinal cord injury and osteoporosis: Causes, mechanisms, and rehabilitation strategies. Int J Phys Med Rehabil (2013) 1:127. 25419534 PMC4238383

[45] Ma Z Yu R Zhao J Sun L Jian L Li C. Constant hypoxia inhibits osteoclast differentiation and bone resorption by regulating phosphorylation of jnk and iκbα. Inflammation Res (2019) 68:157–66. doi: 10.1007/s00011-018-1209-9 30604211

[46] Kang H Yang K Xiao L Guo L Guo C Yan Y. Osteoblast hypoxia-inducible factor-1α pathway activation restrains osteoclastogenesis *Via* the interleukin-33-Microrna-34a-Notch1 pathway. Front Immunol (2017) 8:1312. doi: 10.3389/fimmu.2017.01312 29085370 PMC5650688

[47] Parfitt AM Han ZH Palnitkar S Rao DS Shih MS Nelson D. Effects of ethnicity and age or menopause on osteoblast function, bone mineralization, and osteoid accumulation in iliac bone. J Bone miner Res (1997) 12:1864–73. doi: 10.1359/jbmr.1997.12.11.1864 9383691

[48] Zhao W Li X Peng Y Qin Y Pan J Li J. Sclerostin antibody reverses the severe sublesional bone loss in rats after chronic spinal cord injury. Calcified Tissue Int (2018) 103:443–54. doi: 10.1007/s00223-018-0439-8 PMC7891854 29931461

[49] Ha XQ Yang B Hou HJ Cai XL Xiong WY Wei XP. Protective effect of rhodioloside and bone marrow mesenchymal stem cells infected with hif-1-Expressing adenovirus on acute spinal cord injury. Neural regeneration Res (2020) 15:690–6. doi: 10.4103/1673-5374.266920 PMC6975151 31638093

[50] Komatsu DE Hadjiargyrou M. Activation of the transcription factor hif-1 and its target genes, vegf, ho-1, inos, during fracture repair. Bone (2004) 34:680–8. doi: 10.1016/j.bone.2003.12.024 15050899

[51] Long HQ Li GS Cheng X Xu JH Li FB. Role of hypoxia-induced vegf in blood-spinal cord barrier disruption in chronic spinal cord injury. Chin J traumatol = Zhonghua chuang shang za zhi (2015) 18:293–5. doi: 10.1016/j.cjtee.2015.08.004 26777714

[52] Ding WG Jiang SD Zhang YH Jiang LS Dai LY. Bone loss and impaired fracture healing in spinal cord injured mice. Osteoporosis Int (2011) 22:507–15. doi: 10.1007/s00198-010-1256-8 20445963

[53] Ding WG Yan WH Wei ZX Liu JB. Difference in intraosseous blood vessel volume and number in osteoporotic model mice induced by spinal cord injury and sciatic nerve resection. J Bone miner Metab (2012) 30:400–7. doi: 10.1007/s00774-011-0328-y 22065237

[54] Loboda A Damulewicz M Pyza E Jozkowicz A Dulak J. Role of Nrf2/Ho-1 system in development, oxidative stress response and diseases: An evolutionarily conserved mechanism. Cell Mol Life Sci (2016) 73:3221–47. doi: 10.1007/s00018-016-2223-0 PMC4967105 27100828

