# Supplementary material for: Chronic intermittent hypobaric hypoxia ameliorates osteoporosis after spinal cord injury through balancing osteoblast and osteoclast activities in rats
Source: Front Endocrinol (Lausanne). 2023 May 9;14:1035186. doi: 10.3389/fendo.2023.1035186 (PMC10203702; doi:10.3389/fendo.2023.1035186)
Supplement: Supplementary file 1 [file DataSheet_1.pdf]

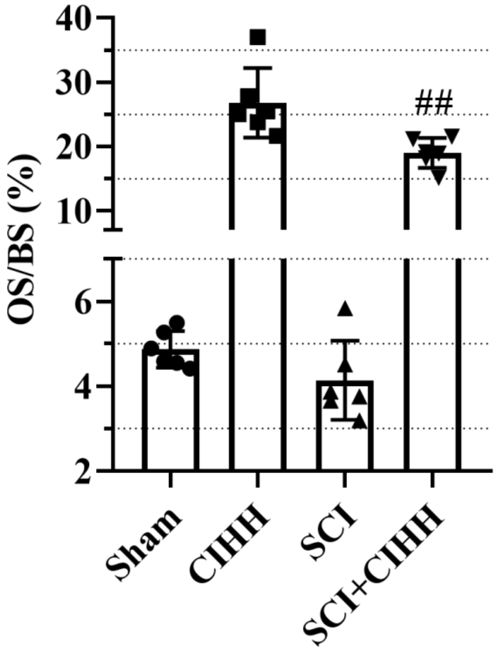

Supplemental Figure 1 Osteoid surface/bone surface (OS/BS) assessed by quantification of Von Kossa staining. Sham: Sham operated group; SCI: Spinal cord injury group; CIHH: CIHH group; SCI+CIHH: SCI plus CIHH group. Data are expressed as mean ± SD. n = 6 in each group. ##p < 0.01 vs SCI.
